# Modifiable antenatal risk factors for stillbirth amongst pregnant women in the Omusati region, Namibia

**DOI:** 10.4102/phcfm.v8i1.1054

**Published:** 2016-05-11

**Authors:** Desire D. Tshibumbu, Julia Blitz

**Affiliations:** 1 Division of Family Medicine, Stellenbosch University, South Africa

## Abstract

**Background:**

Reduction of stillbirth rates is important because of the social and economic implications. Access to quality antenatal care is important in preventing the risk factors associated with stillbirth.

**Aim:**

To determine the prevalence of modifiable antenatal risk factors associated with stillbirth so as to determine possible gaps in their prevention.

**Setting:**

The study was conducted at four district hospitals in the Omusati Region of Namibia.

**Methods:**

A descriptive study using recorded antenatal data was used. Data were collected from the records of 82 women at the time that they had a stillbirth, during the period October 2013 to December 2014. Data were collected for modifiable risk factors related to maternal characteristics, antenatal care received, medical conditions and obstetric complications.

**Results:**

The average prevalence of each category of risk factors was as follows: quality of antenatal care (19.8%), maternal characteristics (11.4%), medical conditions (8.9%) and obstetric complications (6.5%). The most prevalent individual risk factors included: no folate supplementation (30.5%), HIV infection (25.6%), late booking (16.7%), intrauterine foetal growth retardation (13.4%) and alcohol use (12.5%).

**Conclusion:**

Amongst the 14 modifiable risk factor included in the present study, 11 (78.6%) were prevalent amongst women who had a stillbirth. Risk factors associated with quality of antenatal care were the most prevalent. Whilst further investigation is needed to determine the causes behind this prevalence, health education on the availability and benefits of antenatal care, pregnancy timing and spacing may contribute to reducing the prevalence of these risk factors.

## Introduction

The occurrence of stillbirth is usually associated with social and cultural concerns both at a family and country level. At the family level, the death of any child is associated with grief and negative emotions and, at country level, high stillbirth rates add to perinatal mortality rates for the country.^1^ Developing countries are particularly affected and continue to have high stillbirth rates compared with those of the developed world, where a decline has been observed over the last decades. It is estimated that stillbirth rates for developing countries are as high as 25.5 per 1000 deliveries, compared with 5.3 per 1000 deliveries for developed countries.^1^ These rates are a direct reflection of the quality of obstetrical and perinatal care provided in each setting.^1,2^

The quality of care in developing countries is also related to the limited availability of diagnostic resources such as ultrasound, and this has an influence on how stillbirths are classified.^3^ In contrast with developed countries where technological advances have led to many classifications being proposed,^4^ stillbirths in developing countries are commonly categorised based on the dead foetus’ physical appearance as either fresh or macerated.^5^ Fresh stillbirths are recent stillbirths, and the foetus has no decaying skin changes. Macerated stillbirths are deaths that occurred at least 12–24 hours before delivery and the baby has undergone skin changes.^6^ As the present study was conducted in a developing country, the same classification was adopted for the sake of comparison.

It is assumed that risk factors related to both categories of stillbirth are similar in the antenatal period, in contrast with perinatal risk factors, which are often influenced by provider- and resource-related factors.^7,8^ This assumption was considered for the study, and only antenatal risk factors were assessed on whether they were similar in prevalence for either fresh or macerated stillbirths.

The risk factors that were selected for the present study were considered to be potentially modifiable through antenatal programmatic interventions. They included factors related to certain maternal lifestyles, to the quality of antenatal care received, to medical conditions, including infections, and to antenatal obstetric complications (Table 1).

**TABLE 1:** List and definitions of modifiable risk factors included in the study.

Factors	Risk factor	Definition
Factors related to maternal lifestyle	Overweight	Body mass index (BMI) at booking of 25.0 kg/m^2^ or more.^19^
	Underweight	BMI at booking of 18.5 kg/m^2^ or less.^19^
	Alcohol use	The use of alcoholic beverages prior/during this pregnancy.
	Tobacco use	The use of tobacco products prior/during current pregnancy.
Received antenatal
care	Unbooked	Nonattendance of antenatal services prior to delivery.
	Late booking	Start of antenatal visits in the third trimester (gestational age of 28 weeks or more at booking).
	No folate supplementation	Evidence of no supplementation during current pregnancy.
Medical conditions	Positive baseline syphilis	Positive rapid plasma regain at booking.
	HIV infection	Positive HIV test at booking.
	Malaria	Positive malaria test during current pregnancy.
	Uncontrolled pre-existing chronic condition	Pre-pregnancy chronic medical conditions such as hypertension, diabetes and epilepsy, classified according to ICD-10.^13^
Obstetric complications	Pregnancy-induced hypertensive disorders	Either eclampsia or pre-eclampsia, classified according to ICD-10.^13^
	Antepartum haemorrhage	Painless or painful bleeding during current pregnancy.
	Suspected intrauterine foetal growth retardation (IUFGR)†	Birthweight below 10th percentile for gestational age and gender using Williams’ charts.^20^

† Suspected IUFGR was assessed using a California-based chart because there was no agreement on standards for developing countries and because evidence from these countries has shown little difference owing to race or ethnicity when these charts were applied in South Africa and Malawi.^21,22^

These risk factors, and many others, have already been extensively explored in previous studies, and the relationship between the risk factors and stillbirth has been established. However, these studies were conducted mostly in developed settings.^5,6^ The reality in developing countries, especially in Africa, is different, as studies on the topic are inadequate and have often been conducted either in urban areas or in tertiary hospitals,^2,7,8,9^ leaving rural primary healthcare settings almost unexplored. Yet these are the settings where most women in Africa, and in Namibia in particular, access antenatal care services.^10^ It is towards this literature gap that the present study was intended to contribute.

In addition, the study was also intended to assess the prevalence of antenatal risk factors with a view to identifying possible shortcomings that still exist in the implementation of prevention strategies in the four rural districts of the Omusati Region in Namibia. This region’s stillbirth rates have been around 15 per 1000 deliveries in recent years,^11^ with the proportion of macerated stillbirths reaching up to 71% at times. These rates are still a source of concern, and the health authorities aim to reduce them even further. Highlighting the shortcomings and making recommendations about them were a way for this study to contribute to the reduction efforts.

The study objectives were:

1. To determine the prevalence of modifiable antepartum risk factors associated with stillbirth in women of the Omusati Region in Namibia.
2. To make recommendations based on these findings for possible interventions aimed at reducing the prevalence of these risk factors.

## Methods

### Study design

A descriptive, cross-sectional study using recorded data was conducted.

### Study setting

The study was conducted in the four health districts of the Omusati Region, one of the 13 regions of Namibia. The districts are Okahao, Oshikuku, Outapi and Tsandi, and they are generally rural with a combined estimated population of 243 166 people.^12^ The population consists of peasant farmers who access healthcare services through clinics, health centres and district hospitals in the region. Antenatal care services are offered at all levels of healthcare, but most deliveries are conducted at district hospitals and health centres.

### Study population and sampling strategy

The study population consisted of all mothers who gave birth to stillborn babies at the four district hospitals of the Omusati Region during the study period.

Using the total number of deliveries in the Omusati Region in 2013 (5239), a stillbirth rate of 17 per 1000 and a 95% confidence level, a minimum required sample size of 26 was determined using Epi-info StatCalc. As this sample size was small, a decision was made to include all cases of stillbirth during the study period, provided that they met the inclusion criteria.

The inclusion criteria were as follows:

- delivery at one of the four district hospital of the Omusati Region during the study period
- gestational age at time of birth of at least 28 completed weeks as recommended by the World Health Organization for comparison’s sake^13^ and baby weight of at least 700 g, as recommended in Namibia.

The exclusion criteria were as follows:

- twin gestation to avoid confounding owing to its strong association with stillbirth^13^
- stillbirths occurring in the community
- severely incomplete baseline antenatal records.

### Data collection

A record review was conducted from October 2013 to December 2014 on all women who had stillbirths at district hospitals in the region. Data were collected on 14 risk factors (Table 1) from the records of women who had either a fresh or a macerated stillbirth.

A collection tool was developed specifically for the study. It included items to collect data on selected antenatal risk factors routinely assessed during antenatal care in Namibia. The selected risk factors were considered modifiable and previously associated with stillbirth in the literature.^3,9,14,15,16,17,18^ The tool was piloted on 10 women at Tsandi Hospital, and identified shortcomings were corrected.

### Risk factors of interest

The risk factors included are described in Table 1.

### Ethical considerations

Approval to conduct the study was obtained from the Health Research Ethical Committee of Stellenbosch University (reference number S13/08/153) and from the Office of the Permanent Secretary, Ministry of Health and Social Services, Namibia (reference number 17/3/3).

Ethical considerations included the risk of arousing negative emotions in women shortly after a stillbirth experience, minimised by the use of records instead of face-to-face interviews, and the reduced need to obtain written consent from participants after obtaining authorisation to use records. The collected data were anonymised and unique numbers used to identify them.

### Data analysis

Data were analysed using SPSS version 16 and Microsoft Excel 2013. Frequency distributions were calculated for predictor variables (risk factors) and outcome variables (fresh or macerated stillbirth). Comparisons of differences in the prevalence of risk factors between fresh and macerated stillbirth were made to rule out significant differences possibly owing to the influence of perinatal risk factors on fresh stillbirth. Pearson’s chi-squared test or Fisher’s exact test was used as appropriate to determine the significance of these differences at *p* < 0.05. In addition, the relationship between the number of risk factors per woman and some of the most prevalent risk factors was also analysed using one-way independent analysis of variance at *p* < 0.05.

## Results

### Stillbirth rates

During the data collection period of October 2013 to December 2014, there were 8405 deliveries in the Omusati Region. Amongst these, 101 were stillbirths (65 macerated and 36 fresh). This yielded an overall stillbirth rate of 12 per 1000 births, which is lower than in previous years. From these 101 stillbirths, 82 cases (48 macerated stillbirths and 34 fresh stillbirths) were included in the present study because they met the inclusion criteria. The rest were excluded because they met the exclusion criteria.

### Prevalence of modifiable risk factors

**FIGURE 1:**
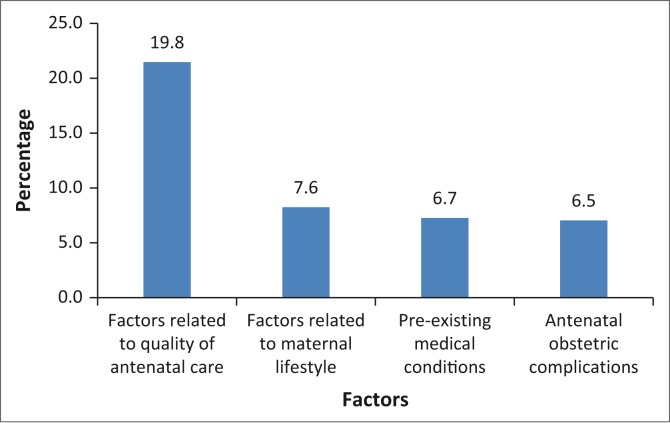
Average prevalence of each group of risk factors.

The average prevalence of each group of risk factors shows that risk factors related to the quality of received antenatal care were the highest at 19.8% (Figure 1).

**FIGURE 2:**
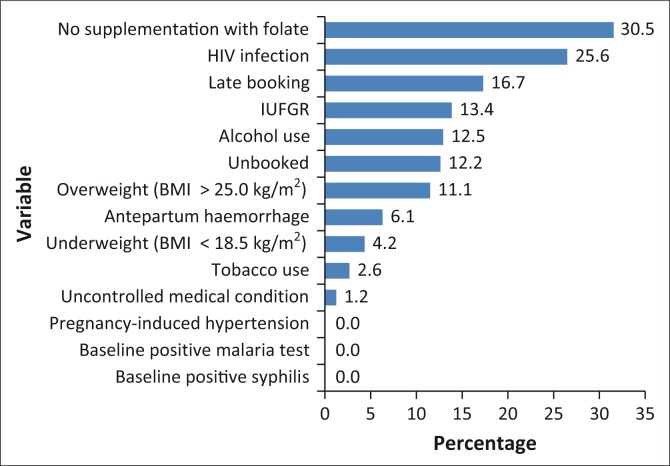
Frequency of each risk factor (*n* = 82).

Further assessment of the prevalence of individual risk factors related to the quality of received antenatal care shows that some women did not receive folate supplements (the most prevalent risk factor at 30.5%), some women started antenatal visits late (16.7%), and some women did not receive any antenatal care because they were unbooked (12.2%) (Figure 2, Table 2). The unbooked mothers were not necessarily those who did not receive folate, as 98.2% of those who did not receive folate attended antenatal care services at least once. The prevalence of no folate supplementation amongst women who had macerated stillbirths was 23.2%, and 7.3% amongst those who had fresh stillbirths. However, this difference was not statistically significant (*p* = 0.051) (Table 2).

**TABLE 2:** Comparison of prevalence between fresh and macerated stillbirths.

Risk factor	Fresh		Macerated		Total		*p*-value
		
	*n*	%	*n*	%	*n*	%	
No folate supplementation (*n* = 82)	6	7.3	19	23.2	25	30.5	0.051
HIV infection (*n* = 82)	8	9.8	13	15.9	21	25.6	0.801
Late booking (*n* = 72)†	8	11.1	4	5.6	12	16.7	0.109
IUFGR (*n* = 82)	5	6.1	6	7.3	11	13.4	1.000‡
Alcohol use (*n* = 80)†	8	10.0	2	2.5	10	12.5	0.013‡
Unbooked (*n* = 82)	3	3.7	7	8.5	10	12.2	0.511‡
Overweight: BMI > 25.0 (*n* = 72)†	3	4.2	5	6.9	8	11.1	1.000‡
Antepartum haemorrhage (*n* = 82)	3	3.7	2	2.4	5	6.1	0.644‡
Underweight: BMI < 18.5 (*n* = 72)†	3	4.2	0	0.0	3	4.2	0.075‡
Tobacco use (*n* = 73)†	1	1.3	1	1.3	2	2.6	1.000‡
Uncontrolled chronic condition (*n* = 82)	1	1.2	0	0.0	1	1.2	0.415‡

Chi-squared test used unless Fisher’s exact test is specified.

† Some records either lacked data or started too late for the data to be used as baseline information;

‡ Fisher’s exact test used owing to violation of assumptions for chi-squared test.

Following risk factors related to the quality of antenatal care, factors related to maternal health related lifestyle were second highest, with an average prevalence of 7.6% (Figure 1). Further examination of the group results revealed that, amongst women who had a stillbirth, 12.5% used alcohol, and 11.1% were overweight. There was no significant difference in prevalence between fresh and macerated stillbirths for this group of factors, with the exception of alcohol use, which was higher in fresh stillbirth cases (*p* = 0.013; odds ratio 0.013).

Risk factors related to medical conditions, including infections, were in third position with an average prevalence of 6.7% (Figure 1). Individual prevalence of risk factors in this group was generally low, with the exception of HIV infection at 25.6% (Figure 2). The apparent difference in HIV prevalence between cases of macerated stillbirths (15.9%) and fresh stillbirths (9.8%) (Table 2) was, however, not statistically significant (*p* = 0.801).

Risk factors related to obstetric complications during the antenatal period were the lowest, with an average prevalence of 6.5% (Figure 1). The most prevalent in this group was intrauterine foetal growth retardation (IUFGR) at 13.4%. It is worth noting that 54.5% of women with IUFGR did not receive folate supplements. Also, 54.5% of them were HIV positive and 9.1% used alcohol.

### Number of risk factors per woman who had a stillbirth

**FIGURE 3:**
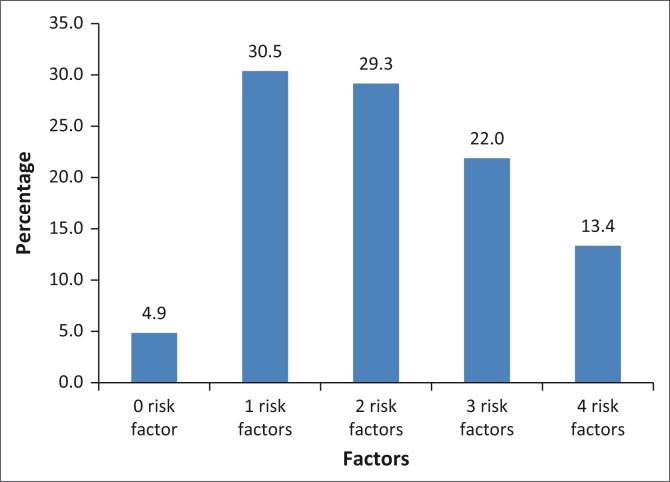
Number of risk factors per woman.

The average number of risk factors per stillbirth was 1.71 (s.d. = 0.91). Only 4.9% of women had none of the 16 risk factors, leaving the remaining 95.1% with a number of factors ranging from one to four (Figure 3). There was a strong association between having a high number of risk factors and HIV infection: *F*(1.80) = 27.42, *p* = 0.00.

## Discussion

The results suggest that risk factors related to the quality of antenatal care received were the most prevalent (19.8%) and that some women received suboptimal antenatal care, either because they were not supplemented with folate (30.5%), they started antenatal visits late (16.7%) or they were unbooked (12.2%). Slightly higher prevalences of no supplementation with folate were reported in West Africa (37.2%)^23^ and in Saudi Arabia (48.6%).^4^ In Nigeria, Fawole reported up to 18.5% of women who were unbooked,^24^ whilst even higher rates were reported in The Gambia (50.0%).^17^

It can be argued that not attending antenatal care services or starting them late could be a woman’s own choice or a consequence of challenges in accessing these services, which is a common occurrence in rural settings. These may be considered as missed opportunities for potentially preventing some stillbirths.

Folate supplementation is recommended by Namibian^25^ and international guidelines.^26^ These recommendations are based on evidence showing folate’s association with reducing the occurrence of neural tube defects, which may eventually lead to stillbirth.^27,28^ Folate is widely available in most health facilities in the region and is provided as a combination pill with iron (Pregamal). According to the findings of the present study, 98.2% of those who visited the health facilities were not supplemented with folate, which raises the question of whether the high prevalence of no supplementation found in this study was because of poor documentation or was a reflection of what actually happens. Findings from the 2013 Namibian Demographic Health Survey (DHS)^10^ which reported up to 22.8% of women not supplemented with folate, support the suspicion of this reality.

The findings also show a substantial proportion of women with risk factors related to their health lifestyle. Amongst these factors were that 12.5% used alcohol and 11.1% were overweight. These factors may be associated either with personal health choices or with a lack of information on making sensible choices concerning these factors, which is common in these settings. Further enquiry is therefore needed to assess the level of knowledge and awareness regarding these risk factors and their prevention. The prevalence of advanced maternal age found in the present study was higher than that found in similar studies in Zambia (29.4%) and South Africa (23%).^7,15^ However, the prevalence of grand multigravidity (17.6%) was consistent with the findings of previous studies in South Africa (21.6%)^7^ and Saudi Arabia (22.5%).^4^

Amongst the medical conditions, one finding of the present study was the high prevalence of HIV infection (25.6%) amongst women who had a stillbirth. This rate is higher than the 17.7% reported by the 2014 Namibia HIV Sentinel Survey for the Omusati Region, or 16.9% for Namibia.^29^ It is not known how many women were on antiretroviral treatment because it was beyond the scope of the study. However, it is worth noting that prevention of mother-to-child transmission interventions at the time of data collection did not include initiation of highly active antiretroviral therapy (HAART) regardless of the level of CD4 count. These changes occurred towards the end of the data collection period, and they were yet to be implemented in all facilities in the region. These were potential missed opportunities because not all women were provided with HAART.

The prevalence of suspected IUFGR was the most notable amongst the risk factors related to antenatal obstetric complications, at 13.4%. As stated earlier, IUFGR was assessed using Williams’ charts^20^ because of limited access to tools such as ultrasound in the region. Early detection could have led to remedial interventions. This was another missed opportunity as some of the districts had ultrasound machines but did not use them because they were not compelled to do so. The examination is not recommended as part of routine antenatal care in the country. This prevalence of IUFGR (19%) is similar to that reported in New Zealand^5^ but lower than in Saudi-Arabia^4^ and Mexico,^30^ at 3.6% and 3.1% respectively.

It is also important to note that the stillbirth rate reported in the present study (12 per 1000 births) appears to be lower than that of recent years for the region (around 15 per 1000). It is relevant that historical stillbirth rates were estimated using data from the regional health information system, which itself may contain some inaccuracies. The other possibility is to regard the reduced stillbirth rate reported as the beginning of a downward trend for the region. This possibility is supported by the fact that an even lower rate was reported by the 2013 DHS (2 per 1000) for the region.^10^ However, this DHS rate is too low and should be interpreted with methodological differences in mind. The DHS used verbal autopsy, which is based on mothers’ recall of previous perinatal deaths.

## Study limitations

The main limitations of the present study include the relatively small sample, owing to the low prevalence of the condition, and the type and quality of the data sources, which were partly from historical records and might have been affected by inaccuracies and omissions.

## Conclusion

The objective of the study was to determine the prevalence of modifiable antepartum risk factors associated with stillbirths in a rural primary health care setting. The findings show that 78.6% of the 14 risks selected for the study factors were prevalent amongst women who had had a stillbirth. The most prevalent group of factors was that related to the quality of received antenatal care, with an average of 19.8%, followed by those related to maternal lifestyle with an average of 7.6%, pre-existing medical conditions at 6.7%, and lastly obstetric complications at 6.5% of the risk factors.

In terms of risk factors related to the quality of received antenatal care, there was, for example, no folate supplementation at 30.5%, late booking at 16.7%, and no booking at 12.2%. The most prevalent factors related to maternal health lifestyle were alcohol use at 12.5%, and overweight at 11.1%. HIV infection at 25.6% was the most prevalent medical condition-related risk factor, whilst undiagnosed IUFGR was the most prevalent obstetric complication-related risk factor at 13.4%.

The prevalence of these risk factors could be reduced and their occurrence could be prevented by providing women with better antenatal education to improve their ability to make informed reproductive health choices and to heighten their awareness of these risk factors.

## Recommendations

Firstly, community awareness and health education on the availability and importance of antenatal care services should be scaled up. This may improve awareness of timing and adherence to prenatal services.

Secondly, an inquiry should be conducted into the reasons for the low uptake of folate supplementation amongst women who access antenatal services. In that way, possible causes may be identified and addressed.

Thirdly, an exploration should be made of the possibility of introducing ultrasound screening at least once during pregnancy for all women attending antenatal care in the region. In that way, foetal wellbeing may be assessed, IUFGR may be detected early and possible supportive measures may be implemented.

Lastly, pre-pregnancy health awareness and education should be enhanced. Women need to be educated about healthy living, alcohol avoidance and pregnancy planning in terms of timing and spacing.

## References

[1] Stanton C, Lawn JE, Rahman H, Wilczynska-Ketende K, Hill K Stillbirth rates: Delivering estimates in 190 countries. Lancet. 2006;367:1487. 16679161 10.1016/S0140-6736(06)68586-3

[2] Mutihir JT, Eka PO Stillbirths at the Jos University Teaching Hospital: Incidence, risk, and etiological factors. Niger J Clin Pract. 2011;14:14–18. 21493985 10.4103/1119-3077.79233

[3] Fretts R Stillbirth epidemiology, risk factors, and opportunities for stillbirth prevention. Clin Obstet Gynecol. 2010;53:588–596. 20661043 10.1097/GRF.0b013e3181eb63fc

[4] Al-Kadri HMF, Tamim HM Factors contributing to intra-uterine fetal death. Arch Gynecol Obstet. 2012;286:1–8. 22714068 10.1007/s00404-012-2426-z

[5] Stacey T, Thompson JM, Mitchell EA, Ekeroma AJ, Zuccollo JM, McCowan LM The Auckland Stillbirth study, a case-control study exploring modifiable risk factors for third trimester stillbirth: Methods and rationale. Aust N Z J Obstet Gynaecol. 2011;51:3–8. 21299501 10.1111/j.1479-828X.2010.01254.x

[6] Mcclure EM, Nalubamba-Phiri M, Goldenberg RL Stillbirth in developing countries.(Report). Int J Gynecol Obstet. 2006;94:82. 10.1016/j.ijgo.2006.03.023 16730726

[7] Ntuli ST, Malangu N An investigation of the stillbirths at a tertiary hospital in Limpopo Province of South Africa. Glob J Health Sci. 2012;4:141. 23121750 10.5539/gjhs.v4n6p141 PMC4776993

[8] Oladapo OT, Adekanle DA, Durojaiye BO Maternal risk factors associated with fetal death during antenatal care in low-resource tertiary hospitals. Aust N Z J Obstet Gynaecol. 2007;47:383–388. 17877595 10.1111/j.1479-828X.2007.00761.x

[9] Feresu SA, Harlow SD, Welch K, Gillespie BW Incidence of and socio-demographic risk factors for stillbirth, preterm birth and low birthweight among Zimbabwean women. Paediatr Perinat Epidemiol. 2004;18:154–163. 14996257 10.1111/j.1365-3016.2003.00539.x

[10] The Nambia Ministry of Health and Social Services (MoHSS), ICF International The 2013 Namibia demographic and health survey [homepage on the Internet]. c2014 [cited 2015 Mar 24]. Available from: http://dhsprogram.com/pubs/pdf/FR298/FR298.pdf

[11] World Health Organization Country stillbirth rates per 1000 total births for 2009 [homepage on the Internet]. c2009 [cited 2013 Mar 3]. Available from: http://www.who.int/pmnch/media/news/2011/stillbirths_countryrates.pdf

[12] Namibia Statistics Agency Namibia 2011 population and housing census indicators. Windhoek, Namibia: Namibia Statistics Agency; 2013.

[13] World Health Organization ICD-10 Version: 2015 [homepage on the Internet]. c2015 [cited 2015 Jun 5]. Available from: http://apps.who.int/classifications/icd10/browse/2015/en

[14] Flenady V, Koopmans L, Middleton P, et al Major risk factors for stillbirth in high-income countries: A systematic review and meta-analysis. Lancet. 2011;377:1331–1340. 21496916 10.1016/S0140-6736(10)62233-7

[15] Stringer EM, Vwalika B, Killam WP, et al Determinants of stillbirth in Zambia. Obstet Gynecol. 2011;117:1151. 21508755 10.1097/AOG.0b013e3182167627

[16] Conde-Agudelo A, Belizán JM, Díaz-Rossello JL Epidemiology of fetal death in Latin America. Acta Obstet Gynecol Scand. 2000;79:371–378. 10830764

[17] Jammeh A, Vangen S, Sundby J Stillbirths in rural hospitals in the Gambia: A cross-sectional retrospective study. Obstet Gynecol Int. 2010;2010:1–8. 10.1155/2010/186867 PMC2910487 20671966

[18] Aminu M, Unkels R, Mdegela M, Utz B, Adaji S, den Broek N Causes of and factors associated with stillbirth in low-and middle-income countries: A systematic literature review. BJOG. 2014;121(s4):141–153. 25236649 10.1111/1471-0528.12995

[19] Waterlow JC Protein-energy malnutrition: The nature and extent of the problem. Clin Nutr. 1997;16(Suppl 1):3–9. 16844615 10.1016/s0261-5614(97)80043-x

[20] Williams RL, Creasy RK, Cunningham GC, Hawes WE, Norris FD, Tashiro M Fetal growth and perinatal viability in California. Obstet Gynecol. 1982;59:624–634. 7070736

[21] van Bogaert L-JJ Customised gravidogram and fetal growth chart in a South African population. Int J Gynecol Obstet. 1999;66:129–136. 10.1016/s0020-7292(99)00068-5 10468335

[22] Verhoeff FH, Brabin BJ, van Buuren S, et al An analysis of intra-uterine growth retardation in rural Malawi. Eur J Clin Nutr. 2001;55:682–689. 11477467 10.1038/sj.ejcn.1601200

[23] Chalumeau M, Bouvier-Colle MH, Breart G, Group M Can clinical risk factors for late stillbirth in West Africa be detected during antenatal care or only during labour? Int J Epidemiol. 2002;31:661–668. 12055171 10.1093/ije/31.3.661

[24] Fawole AO, Shah A, Tongo O, et al Determinants of perinatal mortality in Nigeria. Int J Gynecol Obstet. 2011;114:37–42. 10.1016/j.ijgo.2011.01.013 21489535

[25] The Namibia Ministry of Health and Social Services Namibia standard treatment guidelines [homepage on the Internet]. 2013 [cited 2013 July 7]. Available from: apps.who.int/medicinedocs/documents/s19260en/s19260en.pdf

[26] World Health Organization Guideline: Daily iron and folic acid supplementation in pregnant women. Geneva: World Health Organization; 2012. 23586119

[27] De Regil LM, Fernández-Gaxiola AC, Dowswell T, Peña-Rosas JP Effects and safety of periconceptional folate supplementation for preventing birth defects. Cochrane Database Syst Rev. 2009;(3). 10.1002/14651858.CD007950.pub2 PMC4160020 20927767

[28] De Wals P, Tairou F, Van Allen MI, et al Reduction in neural-tube defects after folic acid fortification in Canada. N Engl J Med. 2007;357:135–142. 17625125 10.1056/NEJMoa067103

[29] Ministry of Health and Social Services Namibia Surveillance report of the 2014 National HIV Sentinel Survey. Windhoek, Namibia: Ministry of Health and Social Services; 2014.

[30] Romero-Gutiérrez G, Martínez-Ceja CA, Abrego-Olvira E, Ponce-Ponce De León AL Multivariate analysis of risk factors for stillbirth in Leon, Mexico. Acta Obstet Gynecol Scand. 2005;84:2. 15603559 10.1111/j.0001-6349.2005.00553.x

